# REUL Is a Novel E3 Ubiquitin Ligase and Stimulator of Retinoic-Acid-Inducible Gene-I

**DOI:** 10.1371/journal.pone.0005760

**Published:** 2009-06-01

**Authors:** Dong Gao, Yong-Kang Yang, Rui-Peng Wang, Xiang Zhou, Fei-Ci Diao, Min-Dian Li, Zhong-He Zhai, Zheng-Fan Jiang, Dan-Ying Chen

**Affiliations:** Key Laboratory of Cell Proliferation and Differentiation (Ministry of Education), College of Life Sciences, Peking University, Beijing, China; Institut de Pharmacologie et de Biologie Structurale CNRS 205, France

## Abstract

RIG-I and MDA5 are cytoplasmic sensors that recognize different species of viral RNAs, leads to activation of the transcription factors IRF3 and NF-κB, which collaborate to induce type I interferons. In this study, we identified REUL, a RING-finger protein, as a specific RIG-I-interacting protein. REUL was associated with RIG-I, but not MDA5, through its PRY and SPRY domains. Overexpression of REUL potently potentiated RIG-I-, but not MDA5-mediated downstream signalling and antiviral activity. In contrast, the RING domain deletion mutant of REUL suppressed Sendai virus (SV)-induced, but not cytoplasmic polyI:C-induced activation of IFN-β promoter. Knockdown of endogenous REUL by RNAi inhibited SV-triggered IFN-β expression, and also increased VSV replication. Full-length RIG-I, but not the CARD domain deletion mutant of RIG-I, underwent ubiquitination induced by REUL. The Lys 154, 164, and 172 residues of the RIG-I CARD domain were critical for efficient REUL-mediated ubiquitination, as well as the ability of RIG-I to induce activation of IFN-β promoter. These findings suggest that REUL is an E3 ubiquitin ligase of RIG-I and specifically stimulates RIG-I-mediated innate antiviral activity.

## Introduction

The innate immune system plays critical roles in recognizing viral infections, and triggers signaling cascades that induce anti-viral mediators such as type I interferons (IFNs) and pro-inflammatory cytokines [1]. Type I IFNs induce the expression of a set of IFN-stimulated genes that inhibit viral replication in infected cells as well as in neighboring uninfected cells. Transcriptional activation of the promoters of type I IFN genes requires the coordinated activation of multiple transcription factors and their cooperative assembly into transcriptional enhancer complexes in vivo. The enhancer of the IFN-β gene contains a κB site recognized by nuclear factor κB (NF-κB), a site for ATF-2/c-Jun, and two IFN-stimulated response elements (ISREs) recognized by phosphorylated interferon regulatory factor (IRF)-3 and/or IRF-7 [2].

The innate immune system has evolved at least two distinct mechanisms for the recognition of viral RNAs [1]. One is mediated by membrane-bound Toll-like receptors, which are important for the production of type I IFNs in plasmacytoid dendritic cells (pDCs). The second mechanism involves cytosolic receptors for RNAs, retinoic-acid-inducible gene-I (RIG-I) and melanoma differentiation associated protein-5 (MDA5), which play essential roles in the recognition of RNA viruses in various cells other than pDCs. Gene-knockout studies indicate that RIG-I and MDA5 are required for responding to distinct species of RNA viruses. RIG-I responds to in vitro-transcribed dsRNA, vesicular stomatitis virus (VSV), Newcastle disease virus (NDV), and influenza virus in mice. In contrast, MDA5 recognizes poly(I∶C) and is essential for the antiviral response to the picornavirus encephalomyocarditis virus [3], [4]. Furthermore, RIG-I, but not MDA5, recognizes single-strand RNA bearing 5′ phosphate [5], [6].

Both RIG-I and MDA5 belong to the DExD/H box RNA helicase family, and contain two CARD modules at their N terminus and a DexD/H-box helicase domain at their C terminus. The helicase domains of RIG-I and MDA5 serve as intracellular viral RNA receptors, whereas the CARD modules are responsible for transmitting signals to the downstream CARD-containing adaptor VISA/MAVS/IPS-1/Cardif, which in turn activates TAK1-IKKα and TBK1/IKKβ kinases, leading to activation of NF-κB and IRF-3 and induction of type I IFNs [7]–[12].

As with other cytokine systems, several mechanisms are thought to underlie the positive and negative regulation of RIG-I signaling. It has recently become clear that RIG-I is regulated by ubiquitin conjugation mediated by a RING finger family protein, RNF125, an E3-ubiquitin ligase that specifies its proteosomal degradation [13]. Expression of RNF125 increases (Lys)-48-linked polyubiquitin and destabilization of RIG-I, while the exact locus for RNF125-mediated RIG-I ubiquitination is not known. Recent studies have shown that polyubiquitination of signaling proteins through lysine (Lys)-63-linked polyubiquitin chains plays an important role in the activation of NF-κB [14], while it was also shown that RIG-I undergoes (Lys)-63-linked ubiquitination at its N-terminal 2CARD. The Lys 172 residue of RIG-I is critical for efficient ubiquitination and for VISA binding, as well as the ability of RIG-I to induce antiviral signal transduction. And the K63-linked ubiquitin is delivered by tripartite motif 25 (TRIM25) E3 ligase [15].

In the present study, we identified a RING-finger protein, REUL, as a novel RIG-I E3 ubiquitin ligase. REUL was specifically associated with RIG-I through its PRY and SPRY domains, and this interaction effectively resulted in a marked increase of RIG-I downstream signaling activity. Furthermore, the Lys 154, 164, and 172 residues of the RIG-I CARD domain were determined to be critical for efficient REUL-mediated ubiquitination and for the ability of RIG-I to induce antiviral signal transduction. These findings suggest that REUL is an E3 ubiquitin ligase of RIG-I and specifically stimulates RIG-I-mediated innate antiviral signaling.

## Results

### Identification of REUL

**Figure 1:**
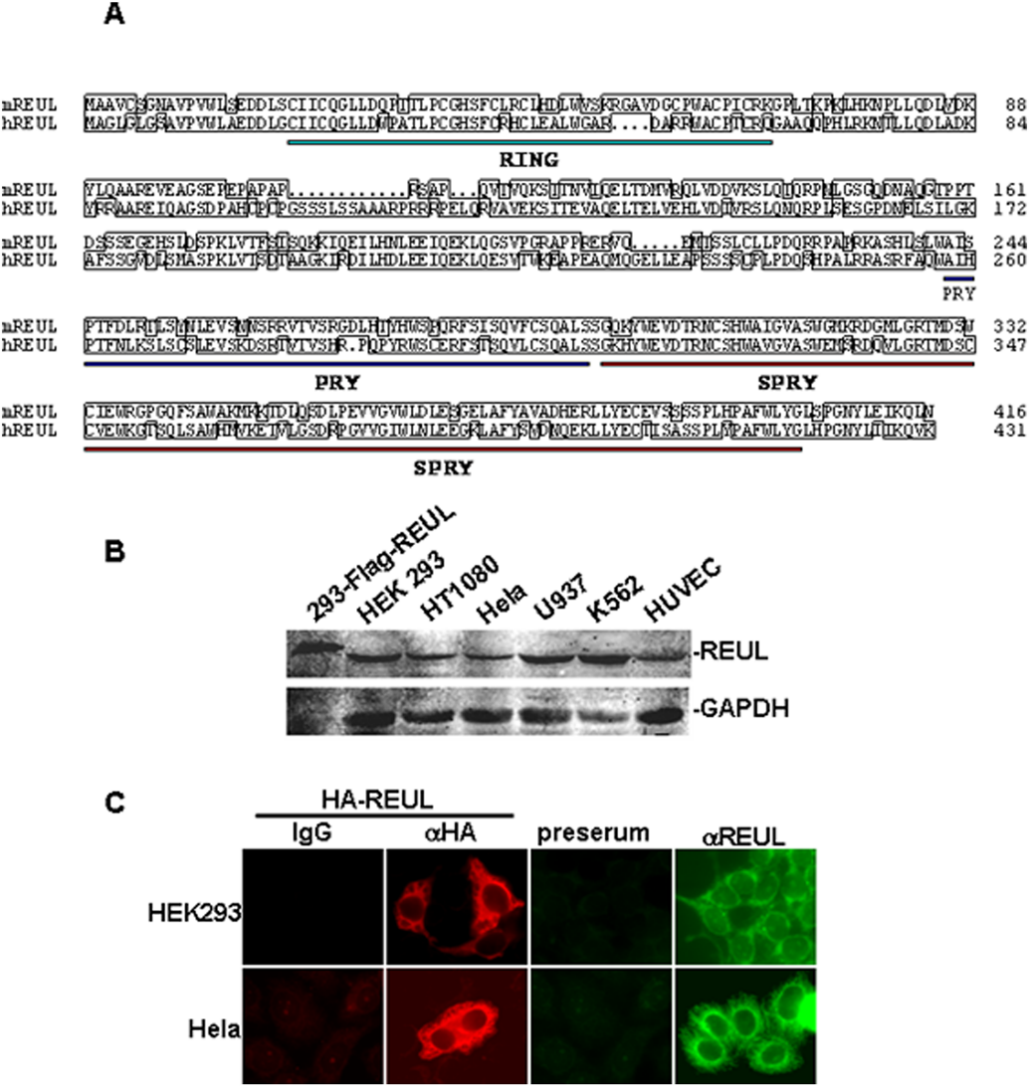
Sequence and expression of REUL. (A) Alignment of human and mouse REUL amino acid sequences. The RING, PRY and SPRY domains are indicated by green, blue and red lines individually. (B) Expression of REUL in mammalian cells. Lysates of the indicated cells were analyzed by Western blot with a mouse polyclonal anti-REUL antibody. (C) REUL localization in HEK293 and Hela cells. Upper panels: HEK293 cells transfected with an expression plasmid for HA-tagged REUL, immunofluorescent staining was performed with anti-HA (αHA) or mouse IgG; untransfected HEK293 cells stained with preimmune serum (preserum) or anti-REUL serum (αREUL); lower panels: transfected or untransfected Hela cells stained as in the upper panels. The experiments were repeated twice, and similar results were obtained.

It is well established that RIG-I is an essential cytosolic sensor for the innate immune response to certain viruses. However, the regulatory mechanism of RIG-I-mediated signaling has not been adequately characterized. To identify potential proteins that interact with RIG-I and regulate its signaling, we performed yeast two-hybrid screens of a human fetal kidney cDNA library using full-length RIG-I as bait. From 5×10^6^ independent screened clones, we identified 17 β-galactosidase-positive clones, among which 5 clones encoded the C-terminal regions of RNF135 (Aa 125–432, GenBank accession number NM_032322.3, and also identified as Riplet in recent publication [16]). This gene is located in a chromosomal region known to be frequently deleted in patients with neurofibromatosis [17]–[19]. On the basis of its functions described below, we designated this protein as REUL (for RIG-I E3 ubiquitin ligase). Since the REUL clone obtained from the yeast two-hybrid screening was not full-length, we amplified full-length REUL cDNA from the human fetal kidney cDNA library using PCR. Human REUL contains 432 amino acid residues and shares 57% sequence identity at the amino acid level with its mouse ortholog (Fig. 1A). Structural analysis with several programs indicated that REUL contains an N terminus RING-finger domain, and a C terminus PRY and SPRY domain (Fig. 1A). The middle region of REUL has no detectable similarity to any other proteins.

To confirm whether REUL is expressed in mammalian cells at the protein level, we raised a mouse antiserum against human REUL. Western blot analysis showed that REUL was expressed as a 48 kDa protein in all examined human cell lines, including HEK293, HT1080, Hela, U937, K562, and HUVEC cells. The size of the endogenous REUL protein was slightly smaller than that of FLAG-tagged REUL overexpressed in HEK293 cells (Fig. 1B). These data suggest that REUL is expressed at the protein level.

We next determined the cellular localization of REUL. Immunofluorescence experiments showed that overexpressed and endogenous REUL were localized in the cytoplasm of HEK293T and Hela cells (Fig. 1C).

### Interaction between RIG-I and REUL

**Figure 2:**
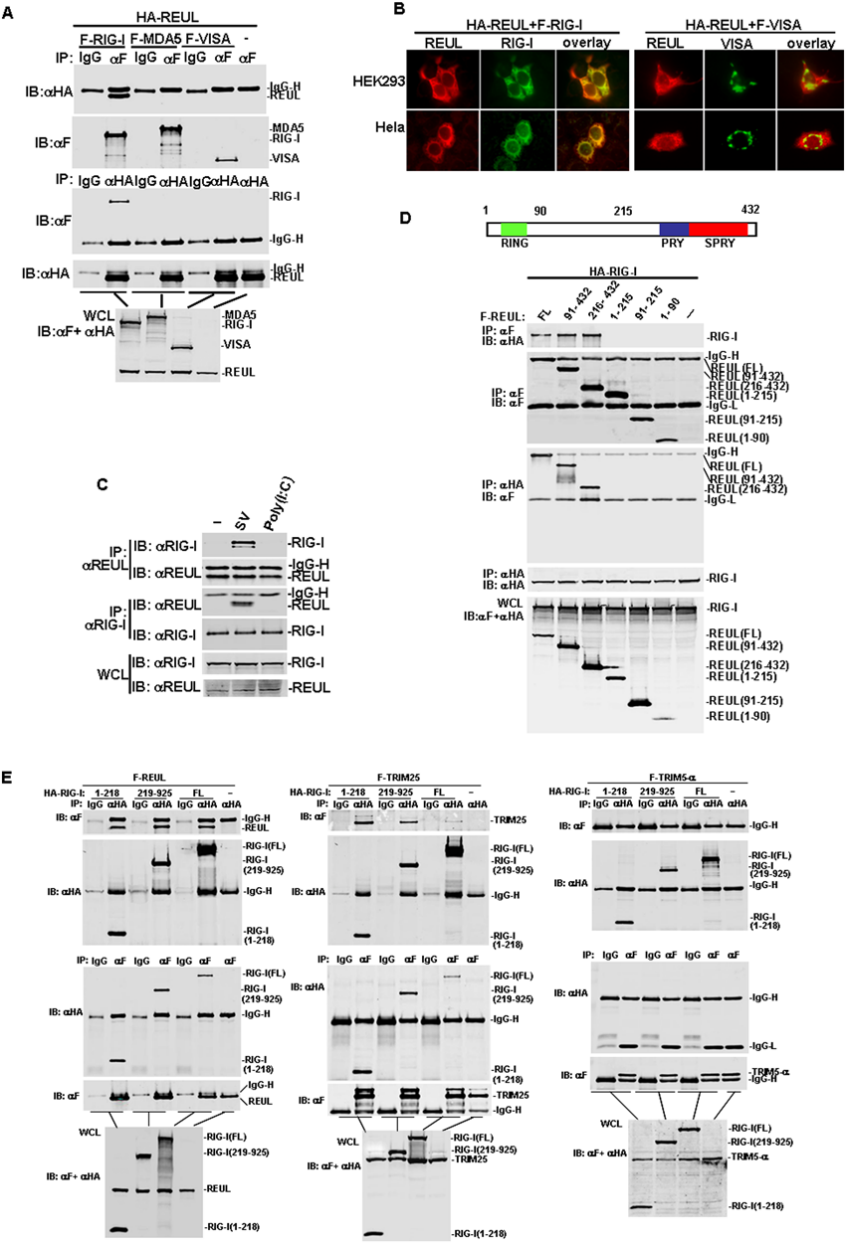
REUL associates with RIG-I. (A) REUL interacts with RIG-I but not MDA5 and VISA. HEK293 cells (1×10^6^) were transfected with HA-REUL alone or together with Flag-RIG-I, Flag-MDA5 and Flag-VISA plasmids (5 µg each). Cell lysates were immunoprecipitated (IP) with anti-Flag (αF), anti-HA (αHA) or mouse IgG antibody (IgG). The immunoprecipitates were analyzed by Western blot (IB) with anti-HA or anti-Flag antibody. Whole-cell lysates (WCL) were analyzed by Western blots with anti-HA and anti-Flag to determine the expression of REUL, RIG-I, MDA5 and VISA. (B) REUL is colocalized with RIG-I but not VISA in HEK293T and Hela cells. Upper panels: HEK293T cells were transfected with HA-REUL, Flag-RIG-I or Flag-VISA. Immunofluorescent staining was performed with anti-HA (red) and anti-Flag (green); lower panels: Hela cells were transfected and stained as in the upper panels. The experiments were repeated twice, and similar results were obtained. (C) Endogenous interaction of REUL and RIG-I. HEK293T (3×10^6^) cells were treated with SV, transfected with polyI∶C (100 µg) or left untreated for 4 h before lysate. Cell lysates were immunoprecipitated (IP) and Western blot (IB) by indicated antibodies. (D) Identification of domains of REUL mediating the interaction with RIG-I. HEK293T cells (1×10^6^) were transfected with expression plasmids for HA-RIG-I alone or together with Flag-REUL or its mutants (5 µg each). Cell lysates were immunoprecipitated with indicated antibodies. The immunoprecipitates were analyzed by Western blots with anti-HA or anti-Flag antibody. Whole-cell lysates were analyzed by Western blots with anti-Flag and anti-HA to determine the expression levels of transfected plasmids. Upper panel: schematic representation of REUL. (E) Identification of domains of RIG-I mediating the interaction with REUL. HEK293T cells (1×10^6^) were transfected with F-REUL (left panel), F-TRIM25 (middle panel), F-TRIM5-α (right panel) alone or together with HA-RIG-I or its mutants. Immunoprecipitation and Western blot analysis were performed with indicated antibodies.

Because REUL interacted with RIG-I in the yeast two-hybrid system, we further determined whether full-length REUL interacted with RIG-I in mammalian cells. Coimmunoprecipitation experiments in HEK293T cells principally indicated that Flag-tagged RIG-I associated with HA-tagged REUL in co-transfection experiments (Fig. 2A). This interaction was specific because in the same experiment REUL did not interact with MDA5, which is structurally and functionally related to RIG-I. REUL also did not interact with adaptor protein VISA (Fig. 2A).

REUL was subsequently shown to colocalize with RIG-I in co-transfected HEK293T and Hela cells (Fig. 2B). Double immunofluorescent staining showed that overexpressed REUL had a similar distribution pattern and overlapped with RIG-I, but not VISA.

We next determined whether REUL interacts with RIG-I in untransfected cells and the effects of viral infection on the interaction. Endogenous coimmunoprecipitation experiments indicated that RIG-I did not interact with REUL without stimulation, but was associated with REUL after SV infection. As a control, REUL did not interact with RIG-I under transfected polyI∶C stimulation (Fig. 2C). These results suggest that REUL is associated with RIG-I in a viral-infection-dependent manner.

REUL contains an N-terminal RING-finger domain, and C-terminal PRY and SPRY domains (Fig. 2D). To determine which domain facilitated interaction with RIG-I, we made REUL deletion mutants. Coimmunoprecipitation assays showed that the mutants carrying PRY and SPRY domains (91–432 and 215–432) bound to RIG-I as effectively as full-length REUL, whereas the RING-finger domain mutant (1–90), the middle region mutant (91–214) and the mutant carrying the RING-finger and the middle region (1–214) did not interact with RIG-I (Fig. 2D). These results suggest that the PRY and SPRY domains of REUL are required for interaction with RIG-I.

RIG-I contains two CARD modules at the N terminus and a DexD/H-box helicase domain at the C terminus. Similarly, we made RIG-I deletion mutants and determined which domain is required for interaction with REUL. Domain-mapping experiments indicated that the mutant carrying the N terminal CARD domain (1–218) and the mutant carrying the DexD/H-box helicase domain (219–925) interacted with REUL individually (Fig. 2E). Since REUL has similar domain structure with TRIM25, we also detected the interactions between TRIM25 and RIG-I truncations for comparison. Coimmunoprecipitation experiments demonstrated that, in the same way, TRIM25 interacted with the helicase domain of RIG-I as efficiently as CARD domain of RIG-I. As control, in the same conditions the RIG-I full length and truncated RIG-I did not interact with TRIM5-α, which has a similar structure to TRIM25 and REUL (Fig. 2E). These results suggest that there are multiple binding sites on RIG-I which mediate the interaction with REUL and TRIM25.

### REUL potentiates RIG-I signaling

**Figure 3:**
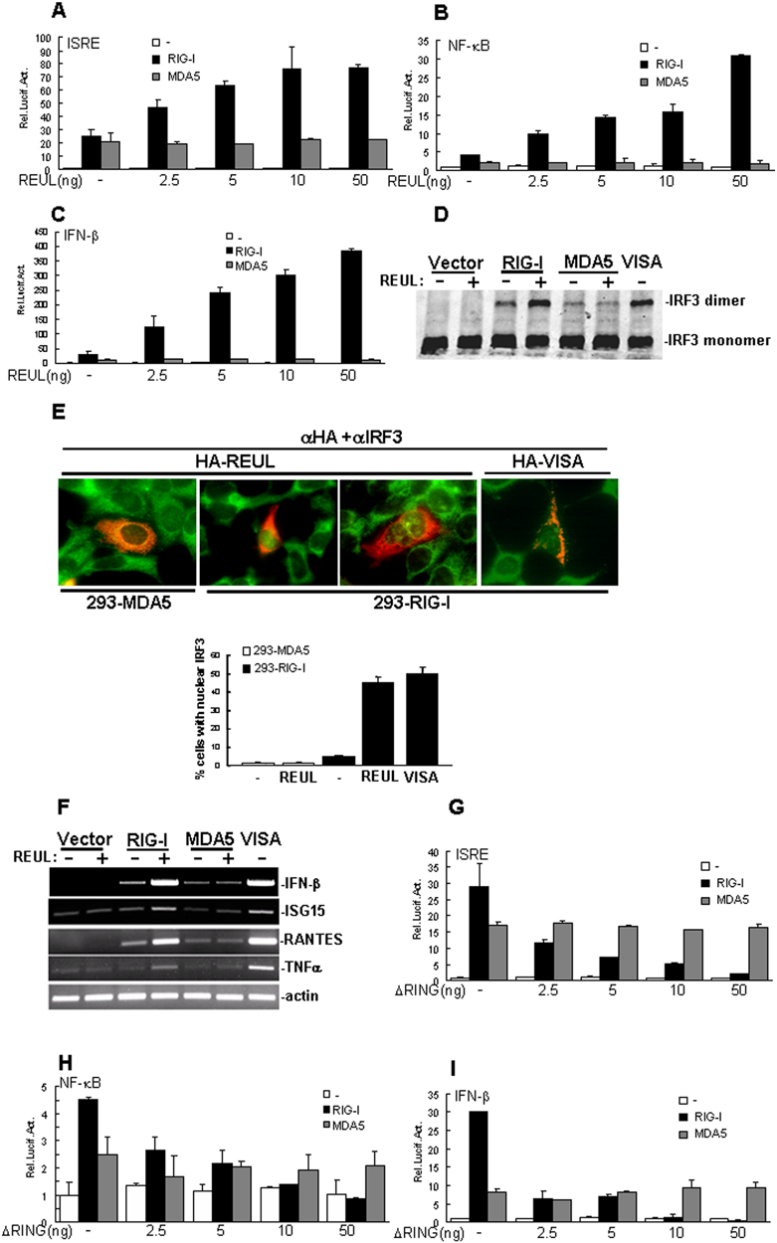
REUL potentiates RIG-I signaling. (A), (B), (C) REUL potentiates RIG-I-, but not MDA5-mediated activation of ISRE (A), NF-κB (B) and IFN-β (C) promoter in a dose-dependent manner. HEK293T cells (1×10^5^) were transfected with the indicated reporter plasmid (50 ng), pRL-TK Renilla luciferase plasmid (50 ng), expression plasmid for RIG-I or MDA5 (200 ng) and the indicated amounts of an expression plasmid for REUL or empty plasmids. Luciferase assays were performed 24 h after transfection. (D) REUL potentiates RIG-I-, but not MDA5-mediated IRF3 dimerization. HEK293 cells (2×10^5^) were transfected with indicated plasmids. 24 h after transfection, cell lysates were separated by native PAGE and analyzed with IRF3 antibody. The experiments were repeated three times with similar results. (E) REUL potentiates RIG-I-, but not MDA5-mediated IRF3 translocation. HEK293-RIG-I stable cell line and HEK293-MDA5 stable cell line (2×10^5^ cells) were transfected with indicated plasmids. 24 h after transfection, immunofluorescent staining was performed with anti-HA antibody (red) and anti-IRF3 antibody (green). The cells expressed REUL or VISA was counted, and the percentage of cells with nuclear IRF3 was calculated. The experiments were repeated twice, and similar results were obtained. (F) REUL potentiates RIG-I-, but not MDA5-mediated gene expression. HEK293T cells (2×10^5^) were transfected with indicated plasmids. 24 h after transfection, total RNA was isolated and RT-PCR was performed using indicated primers. (G), (H), (I) RING domain deletion mutant REULΔRING suppresses RIG-I-, but not MDA5-mediated activation of ISRE (H), NF-κB (I) and IFN-β (G) promoter in a dose-dependent manner. Transfection and luciferase assay were performed as in (A), (B) and (C).

Because REUL is specifically associated with RIG-I, we examined whether REUL is involved in regulation of RIG-I-mediated signaling. In reporter assays, overexpression of REUL alone had no apparent effect on activation of ISRE, NF-κB and IFN-β promoter, while in cotransfection experiments, RIG-I-mediated induction of ISRE, NF-κB and IFN-β promoter activity considerably increased with REUL expression in a dose-dependent manner (Fig. 3A, B, C). In these assays, REUL did not increase MDA5-mediated activation of ISRE, NF-κB and IFN-β promoter (Fig. 3A, B, C).

Dimerization and translocation are hallmarks of IRF3 activation. Consistently, overexpression of REUL potentiated RIG-I- but not MDA5-mediated dimerization of IRF-3 (Fig. 3D). Double immunofluorescence staining showed that overexpression of REUL caused translocation of IRF3 into the nucleus in HEK293 cells stably expressing RIG-I as efficiently as overexpression of VISA, but this did not happen in HEK293 cells stably expressing MDA5 (Fig. 3E).

We further tested whether REUL increased RIG-I-mediated antiviral and pro-inflammatory gene expression. In RT-PCR experiments, we found that cotransfection of REUL potently increased RIG-I-, but not MDA5-induced expression of endogenous antiviral and pro-inflammatory genes, including IFN-β, regulated on activation, normal T-cell expressed and secreted (RANTES), interferon-stimulated gene 15 (ISG15) and TNFα (Fig. 3F). These data suggest that REUL specifically increases RIG-I- but not MDA5-mediated signaling.

Notably, we found that expression of the RING domain deletion mutant REULΔRING (Aa 91–432), which was sufficient to bind to RIG-I, markedly suppressed RIG-I-, but not MDA5-mediated ISRE, NF-κB and IFN-β promoter in a dose-dependent manner (Fig. 3G, H, I). These data suggest that the RING domain of REUL is required for its function to potentiate RIG-I-mediated signaling.

### Role of REUL in RIG-I antiviral activity

**Figure 4:**
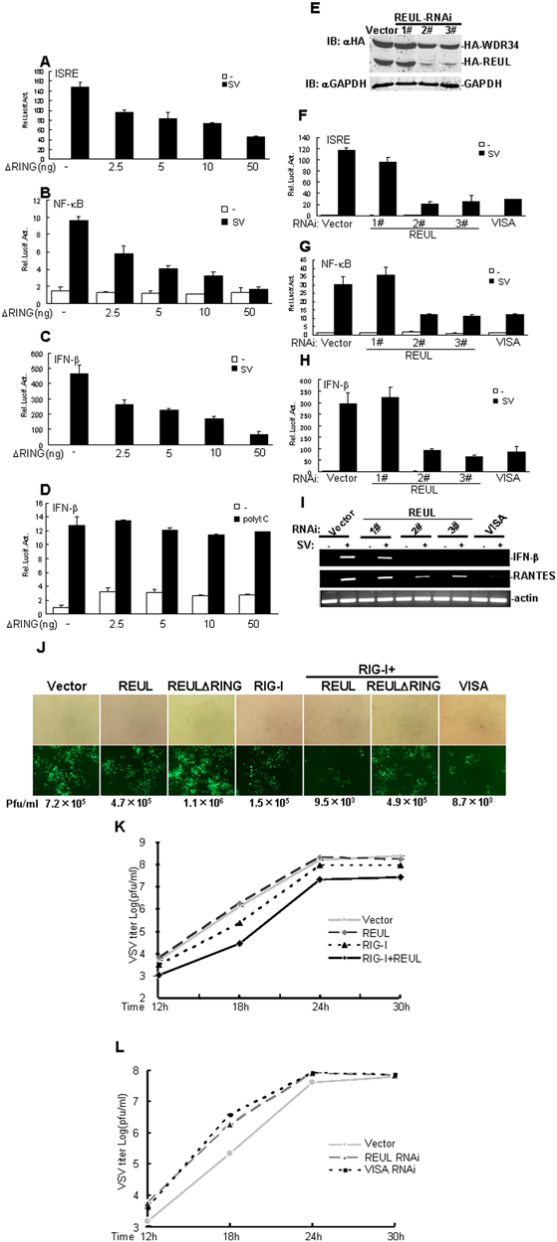
REUL potentiates RIG-I antiviral activity. (A), (B), (C) RING domain deletion mutant REULΔRING suppresses SV-induced activation of ISRE (A), NF-κB (B) and IFN-β (C) promoter in a dose-dependent manner. HEK293T cells (1×10^5^) were transfected with the indicated reporter plasmid (50 ng), pRL-TK Renilla luciferase plasmid (50 ng) and the indicated amounts of an expression plasmid for REULΔRING or empty plasmids. 24 h after transfection, cells were left uninfected or infected with SV for 8 h before luciferase assays were performed. (D) REULΔRING has no effect on polyI:C-induced IFN-β promoter. The transfections were done as in (A), (B) and (C). 16 h after transfection, cells were further transfected with polyI∶C (4 µg) or untransfected for 12 h before luciferase assays were performed. (E) Effects of REUL RNAi plasmids on the expression of transfected REUL. HEK293T cells (2×10^5^) were transfected with expression plasmids for HA-REUL and HA-WDR34 as control (0.5 µg each), and the indicated RNAi plasmids (1 µg). At 48 h after transfection, cell lysates were analyzed by Western blot with anti-HA and anti-GAPDH antibody. (F), (G), (H) REUL RNAi plasmids suppress SV-induced activation of ISRE (F), NF-κB (G) and IFN-β (H) promoter. HEK293T cells (1×10^5^) were transfected with the indicated reporter plasmid (50 ng), pRL-TK Renilla luciferase plasmid (50 ng), and the indicated REUL RNAi (1 µg). At 40 h after transfection, cells were left uninfected or infected with SV for 8 h before luciferase assays were performed. (I) REUL RNAi suppresses RIG-I-, but not MDA5-mediated gene expression. HEK293T cells (2×10^5^) were transfected with indicated plasmids. At 40 h after transfection, cells were left uninfected or infected with SV for 8 h before total RNA was isolated and RT-PCR was performed using indicated primers. (J) REUL suppresses NDV-eGFP replication. HEK293T cells (1×10^5^) were transfected with the indicated plasmids. At 20 h after transfection, cells were infected with NDV-eGFP at MOI 0.001. At 40 h after infection, virus titer and replication were determined by plaque assay or GFP expression visualized by fluorescence microscopy. Pfu, plaque-forming unit. (K) REUL accelerates RIG-I-mediated anti-VSV response. HEK293T cells (2×10^5^) were transfected with indicated plasmids (0.5 µg each). At 30 h after transfection, cells were infected with VSV (MOI = 0.001) and supernatants were harvested at 12, 18, 24 and 30 h post infection. Supernatants were analyzed for VSV production using standard plaque assays. Plaques were counted and titers calculated as plaque-forming units (pfu/ml). (L) Knockdown of REUL inhibits RIG-I-mediated anti-VSV response. The experiments were carried out as in (K).

Previously, it has been demonstrated that RIG-I responds to infection by SV, VSV, NDV, and influenza virus, whereas MDA5 is involved in IFN signaling triggered by poly (I∶C) transfected into the cytoplasm [3], [19].We further determined whether REUL played a role in the cellular antiviral response mediated by RIG-I. In reporter gene assays, overexpression of REULΔRING (Aa 91–432) markedly inhibited SV-induced activation of ISRE, NF-κB and IFN-β promoter in a dose-dependent manner (Fig. 4A, B, C). In contrast, overexpression of REULΔRING had no apparent effect on IFN-β promoter activation triggered by cytoplasmic poly (I∶C) in HEK293T cells (Fig. 4D). These results confirmed that REUL is positively and specifically involved in RIG-I-, but not MDA5-mediated antiviral activity.

We next determined whether endogenous REUL is required for RIG-I-mediated antiviral activity. We utilized three REUL-RNAi plasmids targeting different sites of human REUL mRNA. Transient transfection and Western blot analysis showed that two of these RNAi plasmids (2# and 3#) markedly inhibited the expression of transfected REUL in HEK293T cells, whereas the 1# RNAi plasmid had little effect on REUL expression (Fig. 4E). In reporter gene assays, knockdown of REUL by 2# and 3# suppressed SV-induced activation of ISRE, NF-κB and IFN-β promoter as efficiently as VISA RNAi plasmid, whereas the 1# had no apparent effect on SV-induced signaling (Fig. 4F, G, H). Consistent with this, we found that transfection of REUL RNAi plasmids 2# and 3# potently inhibited SV-induced expression of endogenous IFN-β and RANTES in RT-PCR experiments (Fig. 4I).

We further determined whether REUL plays a role in the cellular antiviral response. On infection by newcastle disease virus -enhanced green fluorescent protein (NDV-eGFP), REUL expression significantly suppressed NDV-eGFP replication in HEK293 cells, whereas expression of REULΔRING detectably increased NDV-eGFP replication. We noted that combined expression of RIG-I and REUL had the strongest effect on suppression of NDV–eGFP replication, and this effection was comparable to overexpression of VISA (Fig. 4J). Using plaque assays, we also found that, cotransfection of REUL efficiently enhanced the inhibitory effect on vesicular stomatitis virus (VSV) replication mediated by overexpression of RIG-I (Fig. 4K), whereas knockdown of REUL had the opposite effect, increasing viral replication as potently as VISA RNAi (Fig. 4L).

Collectively, these data suggest that REUL plays a critical role in the efficient cellular antiviral response mediated by RIG-I.

### REUL is an E3 ubiquitin ligase of RIG-I

**Figure 5:**
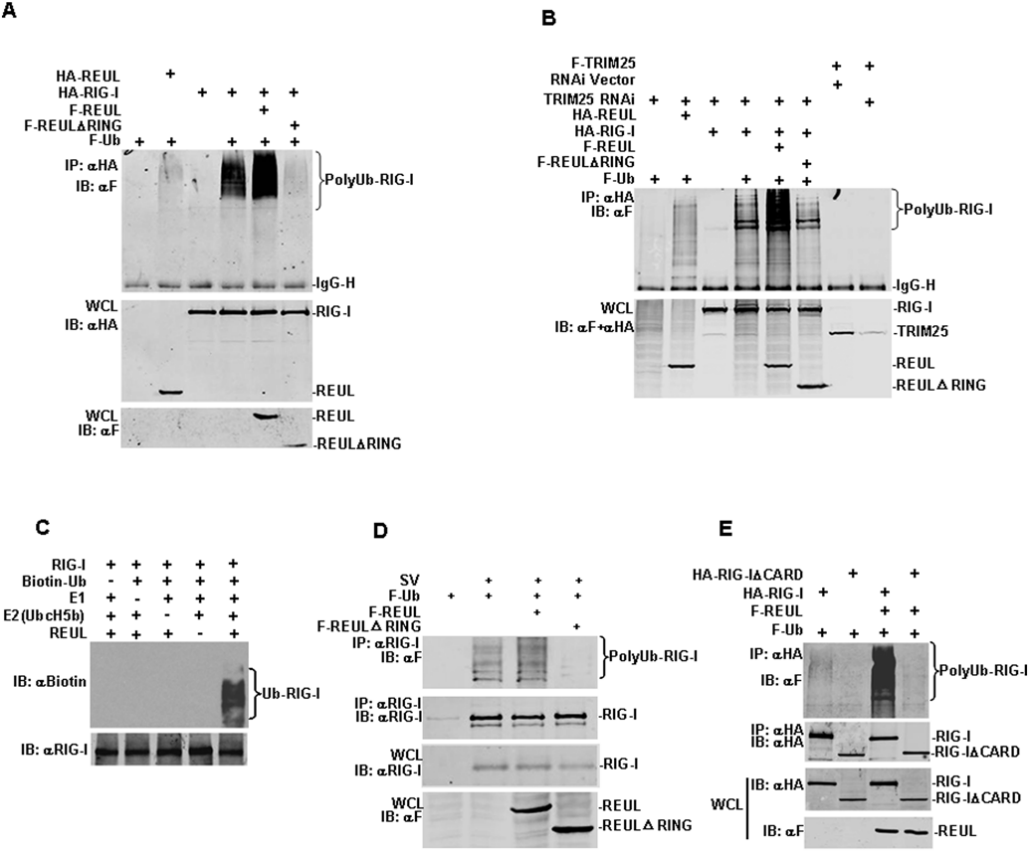
REUL is an E3 ubiquitin ligase of RIG-I. (A) REUL increases the ubiquitination levels of RIG-I in overexpression system. HEK293T cells were transfected with indicated REUL, REULΔRING, RIG-I or ubiquitin plasmids. Cell lysates were immunoprecipitated (IP) with anti-HA (αHA). The immunoprecipitates were analyzed by Western blot (IB) with anti-Flag antibody (αF). Whole-cell lysates were analyzed by Western blots with anti-HA or anti-Flag to determine the expression of REUL, REULΔRING and RIG-I. (B) REUL increases the ubiquitination levels of RIG-I in TRIM25 knock down cells. HEK293T cells were transfected with TRIM25 RNAi and indicated RIG-I, REUL and REULΔRING plasmids. 48 h after transfection, cells were lysated. Immunoprecipitation and western blot were performed as in (A). (C) REUL delivered ubiquitin to RIG-I in vitro. In vitro expressed REUL and RIG-I were used to ubiquitination assay, Biotinylated ubiquitin was detected by HRP-streptavidin. (D) REUL effects on ubiquitination of endogenous RIG-I. HEK293T cells transfected with indicated REUL, REULΔRING, and ubiquitin plasmids. 24 h after transfection, cells were left uninfected or infected with SV for 24 h before lysate. Immunoprecipitation and Western blot were performed as in (A) except that anti-RIG-I antibody (αRIG-I) were used to detect endogenous RIG-I. (E) REUL does not increase the ubiquitination levels of RIG-I ΔCARD. HEK293T cells transfected with indicated REUL, RIG-I, RIG-I ΔCARD and ubiquitin plasmids. Cell lysates were immunoprecipitated with anti-HA (αHA). The immunoprecipitates were analyzed by Western blot with anti-Flag antibody (αF). Whole-cell lysates were analyzed by Western blot with anti-HA or anti-Flag to determine the expression of REUL, RIG-I and RIG-IΔCARD.

Previously, it has been reported that RIG-I undergoes robust ubiquitination at its N-terminal 2CARD, and RIG-I ubiquitination is induced by the TRIM family member TRIM25 in mammalian cells [15]. As the RING domain of REUL is required for potentiation of RIG-I-mediated signaling, we predicted that REUL is a novel E3 ubiquitin ligase of RIG-I. To test the role of REUL in RIG-I ubiquitination, RIG-I was co-expressed with wild-type REUL or the E3 ligase-defective REULΔRING (Aa 91–432) mutant together with ubiquitin. We found that RIG-I was extensively ubiquitinated and REUL expression markedly increased the ubiquitination levels of RIG-I. In contrast, REULΔRING efficiently decreased RIG-I ubiquitination levels (Fig. 5A). To exclude that Trim25 is ultimately affecting RIG-I ubiquitination, we repeated this experiment using Trim25 RNAi and found that in TRIM25 knock-down cells, REUL facilitated the ubiquitination of RIG-I efficiently (Fig. 5B). Finally, *in vitro* ubiquitination assay confirmed that REUL (E3), together with E1 and UbcH5b (E2), effectively delivered the ubiquitin to RIG-I (Fig. 5C). These results demonstrated that REUL directly affects RIG-I ubiquitination, and the effect of REUL is independent of TRIM25.

Furthermore, we detected that SV infection treatment resulted in the markedly increased ubiquitination of endogenously RIG-I. Overexpression of REUL accelerated SV induced ubiquitination of endogenously RIG-I, and REULΔRING potently inhibited this effect (Fig. 5D). These results confirmed REUL effections on ubiquitination of endogenous RIG-I.

To demonstrated which domain of RIG-I was ubiquitinated by REUL, HEK293T cells were co-transfected with Flag-tagged REUL, HA-tagged full-length RIG-I, or a RIG-I mutant RIG-IΔCARD (Aa 219–925), which the 2CARD had been deleted, together with Flag-tagged ubiquitin. REUL expression markedly increased the ubiquitination levels of full-length RIG-I, but not RIG-IΔCARD (Fig. 5E). These data suggested that REUL ubiquitinates the CARD domain of RIG-I.

### The Lys 154, 164, and 172 residues of the RIG-I CARD domain are critical for efficient REUL-mediated ubiquitination and signaling activity of RIG-I

**Figure 6:**
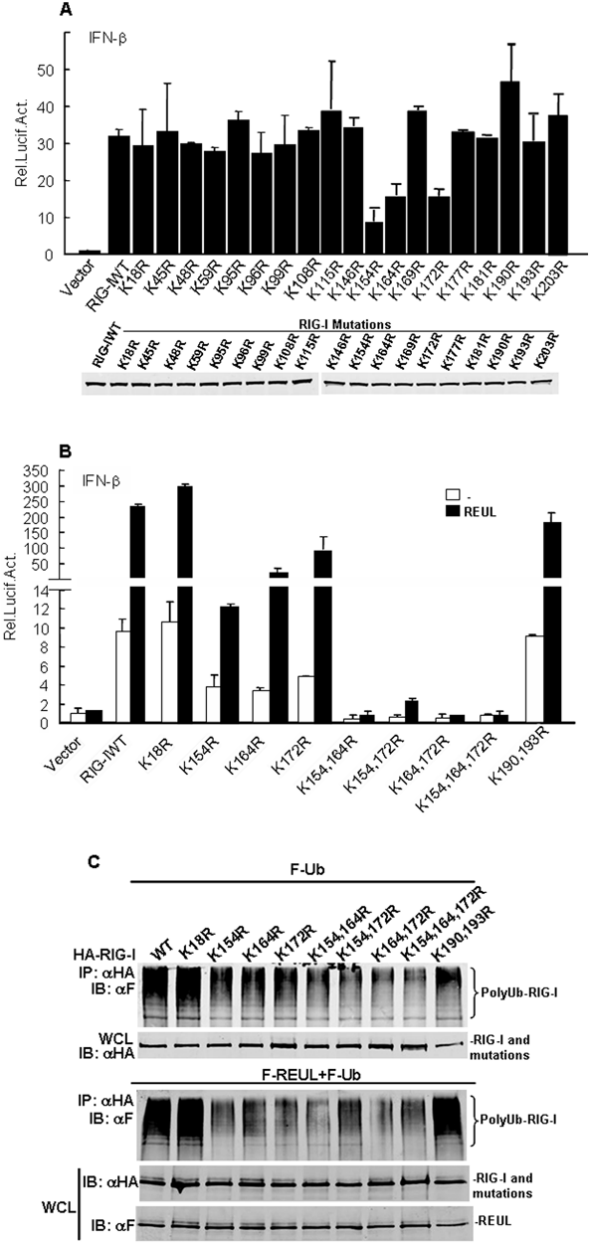
Lys 154, 164, 172 are the essential sites for RIG-I and REUL signaling. (A) K154R, K164R and K172R are the essential sites for RIG-I induced activation of IFN-β promoter. HEK293T cells (1×10^5^) were transfected with IFN-β promoter reporter plasmid (50 ng), pRL-TK Renilla luciferase plasmid (50 ng), and the indicated expression plasmids of RIG-I wild type (WT) and mutations. Reporter assays were performed 24 h after transfection. Lower panel: expression of RIG-I wild type (WT) and mutations. (B) K154R, K164R and K172R are the essential sites for REUL effect. Transfection and luciferase assay were performed as in (A), except cotransfection with REUL. (C) K154R, K164R and K172R are the essential sites for REUL-mediated RIG-I ubiquitination. HEK293T cells were transfected with indicated expression plasmids of RIG-I wild type, mutations and ubiquitin, together with or without REUL expression plasmid. Cell lysates were immunoprecipitated with anti-HA (αHA). The immunoprecipitates (IP) were analyzed by Western blot (IB) with anti-Flag antibody (αF). Whole-cell lysates (WCL) were analyzed by Western blots with anti-HA or anti-Flag to determine the expression of REUL, RIG-I wild type and mutations.

Previously, it has been reported that the Lys 172 residue in the RIG-I CARD domain is critical for TRIM25-mediated ubiquitination and the ability of RIG-I to induce antiviral signal transduction [15]. To further dissect the possible ubiquitination sites of RIG-I that are critically involved in antiviral signaling, we individually replaced the nineteen lysine residues in the RIG-I CARD domain with arginine. In reporter gene assays, we found that compared with wild-type RIG-I, the K154R, K164R and K172R caused marked loss of the ability to induce activation of IFN-β promoter. In contrast, the other KR mutations: K18, 45, 48, 59, 95, 96, 99, 108, 115, 146, 169, 177, 181, 190, 193 and 203R had little or no effect on IFN-β promoter activation (Fig. 6A).

We next tested the functions of mutants K154R, K164R, K172R individually and in various combinations. Reporter assay results showed that the double mutants K164/154R, K172/154R, K172/164R, and the triple mutant K154R/K164R/K172R caused near-complete loss of the ability to induce activation of IFN-β promoter. As control, the K18R and double mutant K190R/K193R induced IFN-β promoter activity as strongly as wild-type RIG-I (Fig. 6B). These data suggested that Lys 154, 164 and 172 are the critical sites for RIG-I signaling activity.

We also determined whether Lys 154, 164, and 172 are the key sites for REUL-mediated ubiquitination and activity of RIG-I. Reporter assay results showed that overexpression of REUL potently potentiated the ability of wild-type RIG-I to activate IFN-β promoter. But the K154R, K164R and K172R mutations significantly reduced the activation level, and REUL almost completely failed to induce activation of RIG signaling when cotransfected with the double mutants K164/154R, K172/154R, or K172/164R, or the triple mutant K154R/K164R/K172R (Fig. 6B). In contrast, co-expression with REUL, K18R and K190R/193R induced IFN-β activity as strongly as the wild-type. These data indicated that Lys 154, 164, and 172 are the key sites for the REUL-mediated signaling activity of RIG-I.

To test the role of Lys 154, 164, and 172 in RIG-I ubiquitination mediated by REUL, wild type RIG-I and mutations were co-expressed with or without REUL. Consistently, with REUL expression, the ubiquitination levels of wild type RIG-I, K18R and K190R/193R were markedly increased, whereas those of K154R, K164R, or K172R, the double mutants K164/154R, K172/154R, K172/164R, or the triple mutant K154R/K164R/K172R were minimal. We also detected the similar result without REUL expression (Fig. 6C). These data suggest that Lys 154, 164, and 172 are the primary sites for efficient REUL-mediated ubiquitination of RIG-I.

## Discussion

REUL has also been identified as Riplet in a recent publication [20]. Riplet has been reported to promote RIG-I-mediated interferon-β promoter activation, and inhibit propagation of a negative-strand RNA virus (vesicular stomatitis virus). Our results confirmed the functions of this protein. But there are several critical differences between the findings for REUL and Riplet. Firstly, Riplet was reported to promote lysine 63-linked polyubiquitination of the C-terminal region of RIG-I, which modification was considered by the authors to differ from the N-terminal ubiquitination by TRIM25 [16]. But in our study, no apparent ubiquitination level was detected in overexpression of RIG-IΔCARD (carrying helicase and RD domains) alone or together with REUL. As a control, in the same conditions, full-length RIG-I was extensively ubiquitinated and REUL expression markedly increased the ubiquitination levels of RIG-I. These results suggested that the CARD domain, but not the helicase domain of RIG-I is ubiquitinated through REUL action.

Secondly, Riplet has been reported to bind to the C-terminal helicase and RD regions of RIG-I, and fails to co-precipitate the CARD domains of RIG-I [16]. In our study, the CARD and helicase domains of RIG-I interacted with REUL individually, and efficiently as full-length RIG-I. This result suggested that there are multiple binding sites on RIG-I which mediate the interaction with REUL.

Furthermore, Riplet has been reported to potentiate polyI:C-induced activation (cells stimulated with medium containing polyI∶C) of the IFN-β promoter [16]. In our study, polyI∶C did not activate IFN-β expression in TLR3-deficient 293T cells when applied extracellularly (data not shown). And REUL or REULΔRING also had no effect on signaling induced by intracellular (transfected) polyI∶C in HEK293T cells. Previously, it has been demonstrated that, in mouse embryonic fibroblasts, MDA5 is involved in IFN induction triggered by polyI∶C transfected into the cytoplasm [3]. And in reporter assays, knockdown of MDA5 significantly inhibits cytoplasmic polyI:C-induced ISRE activation, whereas knockdown of RIG-I has a minimal inhibitory effect [19], suggesting that cytoplasmic polyI:C-induced IRF-3 activation is mostly mediated by MDA5 in HEK293 cells. In our study, REUL was associated with RIG-I but not MDA5 in mammalian cells (Fig. 2). Overexpression of REUL inhibited RIG-I- but not MDA5-mediated activation of ISRE, NF-κB and IFN-β promoter (Fig. 3). Overexpression of REULΔRING efficiently inhibited SV, but not polyI:C-induced IFN-β promoter activation in HEK293T cells (Fig. 4). These results confirm that REUL is specifically involved in the RIG-I-, but not the MDA5-mediated signaling pathway.

Another E3 ligase, Trim25, has recently been shown to induce K63-linked ubiquitination of RIG-I and to contribute to the activation of downstream signaling pathways [15]. TRIM25 and REUL have homologous domain patterns and regulate RIG-I in a similar manner. Both of them deliver ubiquitin to the CARD domain of RIG-I. In addition, REUL interacts with RIG-I through its C-terminal PRY and SPRY domains; this is the same as TRIM25, which also contains a C-terminal SPRY domain. On the other hand, the CARD domain of RIG-I has been reported to bind TRIM25, and the interaction between the helicase domain and TRIM25 has not been identified [15]. In our study, we found that in the same way, REUL and TRIM25 interacted with the helicase domain as well as the CARD domain of RIG-I. These results suggested that there are multiple binding sites on RIG-I which mediate the interaction with REUL and TRIM25, although only the CARD domain is their ubiquitination target.

The Lys 172 residue of RIG-I is critical for efficient TRIM25-mediated ubiquitination and for VISA binding [15], as well as the ability of RIG-I to induce antiviral signal transduction. In the same report, mutations of K99, 169, 181, 190 and 193 were demonstrated to induce IFN-β and NF-κB promoter efficiently. Our study confirmed these results and notably suggested that Lys 154 and Lys 164 are also critical sites for RIG signaling. The K154R and K164R mutants disrupted the signaling function of RIG-I to induce activation of IFN-β promoter as strongly as the K172R mutant. Combined double or triple mutants caused near-complete loss of the ability of RIG-I to induce activation of IFN-β promoter. Our results showed that not only K172, but also K154 and K164 are involved in REUL-mediated RIG-I ubiquitination and signaling.

Although TRIM25 and REUL regulate RIG-I in a similar manner, they function independently of each other. Using Trim25 siRNA, we found that in TRIM25 knock-down cells, REUL facilitated the ubiquitination of RIG-I efficiently. And in vitro ubiquitin assays showed that REUL directly delivered ubiquitin to RIG-I. These results demonstrated that the function of REUL is independent of TRIM25. TRIM25 and REUL are not ubiquitously distributed over the organs tested. Northern blots showed that human Riplet is expressed in human skeletal muscle, spleen, kidney, placenta, prostate, stomach, thyroid and tongue, and weakly expressed in heart, thymus, liver, lung and uterus [16]. Whereas TRIM25/Efp is predominantly expressed in various female organs such as the uterus, ovary, mammary gland and placenta [20]. Since TRIM25 and REUL have different distribution patterns, we suggest that REUL may be a complement factor of TRIM25 for RIG-I activation.

We noted that expression of the mutant with the RING domain deleted, REULΔRING, markedly suppressed RIG-I-mediated and SV-induced ISRE, NF-κB and IFN-β promoter, and also efficiently inhibited NDV replication and RIG-I ubiquitination. These data indicate that, as a dominant negative mutant, REULΔRING competes with endogenous REUL to bind with and ubiquitinate RIG-I, and block RIG-I signaling.

As a cytosolic receptor, RIG-I activity is tightly regulated by several mechanisms. LGP2, a RNA helicase protein lacking CARD domains at its N terminus, acts as a negative regulator by sequestering viral RNA from RIG-I [21], [22]. CYLD targets RIG-I for deubiquitination that leads to inactivation of the signaling [23], [24]. The autophagic protein conjugate Atg5-Atg12 negatively regulates the type I IFN production pathway by direct association with the CARD domain of RIG-I [25]. ISG15 has been reported to modify RIG-I and act as a negative feedback regulator [26]. Two RING finger proteins, RNF125 and TRIM25, have been shown to play critical roles in regulating RIG-I activity as E3 ubiquitin ligase. RNF125-mediated K48-linked polyubiquitination leads to the degradation of RIG-I [13], and TRIM25-mediated K63-linked polyubiquitination activates RIG-I [15]. In the present work, we identified REUL as a novel RIG-I E3 ubiquitin ligase that specifically potentiates RIG-I-mediated antiviral activity. This finding extends the regulator family of RIG-I, and the mechanisms underlying this finding deserve further study, especially using knockout techniques.

## Materials and Methods

### Reagents and cell lines

Mouse monoclonal antibodies against Flag and HA epitopes (Sigma, USA), poly(I∶C) (InvivoGen, USA), IRDye800-conjugated anti-mouse and anti-rabbit IgG (Rockland Immunochemicals, USA), mouse monoclonal antibody to RIG-I (Alexis Biochemicals, Switzerland), rabbit polyclonal antibodies against IRF3 (SC-9082), SV (Hong-Bing Shu, Wuhan University, Wuhan, China), NDV-eGFP (Cheng Wang, Institute of Biochemistry and Cell Biology, Shanghai, China) and VSV (Hong-Kui Deng, Peking University, Beijing, China) were obtained from the indicated sources. Mouse anti-REUL antibody was prepared with recombinant protein by Experimental Animal Center, Institute of Genetics and Developmental Biology, Chinese Academy of Sciences. HEK293, BHK21, HT1080, Hela, HUVEC and U937 cells were grown in DMEM supplemented with 10% fetal bovine serum. K562 cells were grown in IMDM supplemented with 10% fetal bovine serum.

### Yeast Two-Hybrid Screens

The human fetal kidney cDNA library (Clontech, USA) was screened with full-length RIG-I as bait, following protocols recommended by the manufacturer.

### Constructs

Mammalian expression plasmids for Flag- or HA-tagged TRIM25, TRIM-5α, RIG-I, REUL, and their deletion mutants were constructed by standard molecular biology techniques. Mammalian expression plasmids for Flag-VISA, Flag-MDA5, Flag-Ub, and ISRE, NF-κB, and IFN-β promoter luciferase reporter plasmids were kindly provided by Dr. Hong-Bing Shu (Wuhan University, Wuhan, China).

### Transfection and Luciferase Assays

HEK293T cells were seeded on 24-well dishes and transfected the next day by standard calcium phosphate precipitation. In the same experiment, we added empty control plasmid to ensure that each transfection received the same amount of total DNA. To normalize for transfection efficiency, 0.05 µg of pRL-TK (Renilla luciferase) reporter plasmid was added to each transfection. Luciferase assays were performed using a dual-specific luciferase assay kit (Promega, USA). Firefly luciferase activity was normalized based on Renilla luciferase activity. All reporter assays were repeated at least three times. Data shown are average values ±SD from one representative experiment.

### Coimmunoprecipitation and Western Blot Analysis

For transient transfection and immunoprecipitation experiments, HEK293T cells (∼2×10^5^) were transfected with the indicated plasmids for 20 h. The transfected cells were lysed in 0.5 ml of lysis buffer (20 mM Tris, pH 7.5, 150 mM NaCl, 1% Triton X-100, 1 mM EDTA, 10 µg/ml aprotinin, 10 µg/ml leupeptin, 1 mM phenylmethylsulfonyl fluoride). For each immunoprecipitation, a 0.4 ml aliquot of lysate was incubated with 0.5 µg of the indicated antibody and 25 µl of a 1∶1 slurry of protein-A sepharose (GE Healthcare, USA) for 2 h. The sepharose beads were washed three times with 1 ml of lysis buffer. The precipitates were analyzed by Western blot with the indicated antibodies and visualized by incubation with IRDye800-conjugated secondary antibodies (diluted 1∶10,000) using an Odyssey infrared imaging system (LICOR Inc., Germany).

### Native PAGE

Native PAGE (8%) was pre-run for 30 min at 40 mA with native running buffer (25 mM Tris and 192 mM glycine, pH 8.4) and with 0.5% deoxycholate in the cathode chamber. Samples were prepared in the native sample buffer (62.5 mM Tris–HCl, pH 6.8, 15% glycerol and 0.5% deoxycholate).

### Immunofluorescent staining

Cells were fixed with ice-cold methanol for 10 min at −20°C, rehydrated three times with PBS, and blocked in 5% bovine serum albumin/PBS for 10 min. The cells were stained with primary antibody in blocking buffer for 1 h at 37°C, rinsed with PBS, and stained again with FITC-conjugated Affinipure rabbit anti-mouse IgG or Texas Red-conjugated Affinipure goat anti-rabbit IgG (1∶200 dilutions) for 1 h at 37°C. The cells were then rinsed with PBS containing DAPI and mounted in Prolong Antifade (Molecular Probes). The cells were observed under an Olympus BX51 immunofluorescence microscope using a ×100 plan objective.

### In vitro Ubiquitinlation Assay

In vitro protein expression was performed using TNT® quick coupled transcription/translation systems (Promega, USA), Ubiquitinlation Assay was performed using ubiquitinylation kit (ENZO life sciences, USA), Following protocols recommended by the manufacturer.

### RNAi Experiments

Double-strand oligonucleotides corresponding to the target sequences were cloned into the pSuper.retro RNAi plasmid (Oligoengine, USA). In this study, the target sequences for human REUL cDNA were 1#: 5′AACATCTTGTAGACATTGTCA 3′; 2#: 5′AATCAGAGATATTCTCCATGA 3′; 3#:5′AAGTGGACACTAGGAATTGCA 3′; positive control VISA RNAi target sequence 5′GTATATCTGCCGCAATTTC 3′.

### RT-PCR

Total RNA was isolated from HEK293T cells by using TRIzol reagent (Tianwei Co., Beijing, China) and subjected to RT-PCR analysis to measure expression of IFN-β, ISG15, RANTES, TNFα and β-actin. The gene-specific primer sequences were: IFN-β, sense: 5′-CCAACAAGTGTCTCCTCCAA-3′, antisense: 5′-ATAGTCTCATTCCAGCCAGT-3′; ISG15, sense: 5′-GCTGGGACCTGACGGTGAAGA-3′, anti-sense: 5′-GCTCAGAGGTTCGTCGCATTTGT-3′; RANTES, sense: 5′-CCTCGCTGTCATCCTCATTG-3′, anti-sense: 5′-TACTCCCGAACCCATTTCTT-3′; TNFα, sense: 5′-CCTGGTATGAGCCCATCTATC-3′, antisense: 5′-CGAAGTGGTGGTCTTGTTGC-3′; β-Actin, sense: 5′-ACGTGGACATCCGCAAAGAC-3′, antisense:5′-CAAGAAAGGGTGTAACGCAACTA-3′.

### VSV plaque assay

HEK293T cells (∼1×10^5^) were transfected with the indicated plasmids for 24 h prior to VSV infection. At 1 h post-infection, cells were washed with PBS and then fresh medium was added. The supernatant was harvested at the indicated times and used to infect confluent BHK21 cells cultured on 24-well plates. At 1 h post-infection, supernatant was removed and culture medium containing 2% methylcellulose was overlaid. At 60 h post-infection, the overlaid medium was removed; cells were fixed with 0.5% glutaraldehyde for 30 min and stained with 1% crystal violet dissolved in 70% ethanol. Plaques were counted, averaged and multiplied by the dilution factor to determine viral titer as Pfu/ml. The experiments were repeated three times and each experiment was performed in duplicate. Data shown are average values ±S.D. from one representative experiment.
